# Results from a cervical cancer screening program in Samsun, Turkey

**DOI:** 10.1186/s12905-022-01916-6

**Published:** 2022-08-04

**Authors:** Hatice Nilden Arslan, Muhammet Ali Oruc

**Affiliations:** 1 Department of Public Health, Faculty of Medicine, Ondokuz Mayis University, Samsun, Turkey; 2 Department of Family Medicine, Faculty of Medicine, Ahi Evran University, Kirsehir, Turkey

**Keywords:** HPV, Cervical cancer, Cancer screening, Women’s health

## Abstract

**Background:**

Cervical cancer is a preventable disease. This study aimed to share the results of the national cervical cancer screening program performed in primary health care institutions in Samsun between 2015 and 2019.

**Methods:**

Women aged 30–65 years who were screened for cervical cancer in screening centers of Samsun between January 01, 2015, and December 31, 2019, were included in this descriptive study. The data were obtained from the automation program of the “National Human Papilloma Virus (HPV) Laboratory Application” used by the Provincial Directorate of Health Cancer Unit through filtering the completion time of the tests, and all results were evaluated without sampling. Thus, data were presented using descriptive statistics.

**Results:**

The mean age of 89,302 women included in the cervical cancer screening program was 45.9 ± 9.0 years. Of the samples obtained from the participants, 1.0% were determined as insufficient material, 94.1% as HPV-negative, and 4.9% as HPV-positive. The most common HPV genotypes were 16, 51, 31, and 52. Of the 4337 HPV-positive women, 74.7% of the pap smear results were negative (including infection, 36.5%), and the most common premalignant lesions were atypical squamous cells of undetermined significance in 7.1% and low-grade squamous intraepithelial lesions in 6.9%. HPV 16/18 was also observed in 31.7% of HPV-positive women. Seven hundred ninety-five women were referred to a specialist physician for further examination and treatment within the scope of the screening algorithm.

**Conclusion:**

Detecting HPV-positivity by reaching more women within the national cervical cancer screening program’s scope is vital in fighting against this disease. The effectiveness of cancer screening programs should be increased by ensuring community participation through awareness activities.

## Background

Cervical cancer is one of the leading causes of cancer death among women. According to the data of 2018 from the World Health Organization (WHO), cervical cancer is the fourth most common type of cancer among women after breast, colorectal, and lung cancer. While approximately 570,000 women have cervical cancer every year worldwide, 311,000 women die due to this cancer, and 88% of these deaths occur in low-income countries [1]. Cervical cancer is the 9th most common cancer among women in Turkey and constitutes 2.4% of all age groups in women.[2].

The most important cause of cervical cancer is type 16, 18, one of the high-risk oncogenic Human Papilloma Virus (HPV) types, and almost all cervical cancer cases develop due to HPV infection. HPV infection is one of the most frequent sexually transmitted diseases. HPV lesions in the genital system are common in some age groups, as most regress spontaneously and are asymptomatic [3]. However, it can still lead to clinical diseases such as genital HPV infection, anogenital warts, cervical neoplasia, cervical cancer, and other anogenital cancers [4]. HPV has more than 100 subtypes, and at least 14 cause cancer. Screening is vital since the development of cervical cancer following HPV infection may take up to 15–20 years in women with normal immune systems. Therefore, in 2018, the WHO decided to eradicate cervical cancer and emphasized that vaccination against HPV and appropriate treatment of HPV-positive women determined in screening tests would reduce deaths due to this cancer [5]. In its global call on November 17, 2020, WHO launched the “Cervical Cancer Elimination Program” and committed to eliminating a type of cancer for the first time. Within the scope of this program, countries were asked to take action to achieve the following objectives. To eliminate cervical cancer, all countries must reach and maintain an incidence rate of below four per 100,000 women. Achieving that goal rests on three key pillars and their corresponding targets:

- Vaccination: 90% of girls being fully vaccinated with the HPV vaccine by the age of 15;
- Screening: 70% of women being screened using a high-performance test by the age of 35 and again by the age of 45;
- Treatment: 90% of women with pre-cancer being treated, and 90% of women with invasive cancer being managed [6].

Thus, detecting the disease at an early stage and removing precursor lesions, such as cervical intraepithelial neoplasia (CIN), will reduce invasive cervical cancer cases [7]. Cervical intraepithelial neoplasia (CIN) is a premalignant lesion with three stages, CIN1, CIN2, and CIN3, and it can be detected with current screening tests with the “Scan and treat” approach. Therefore, appropriate treatment can be ensured.

**Fig. 1:**
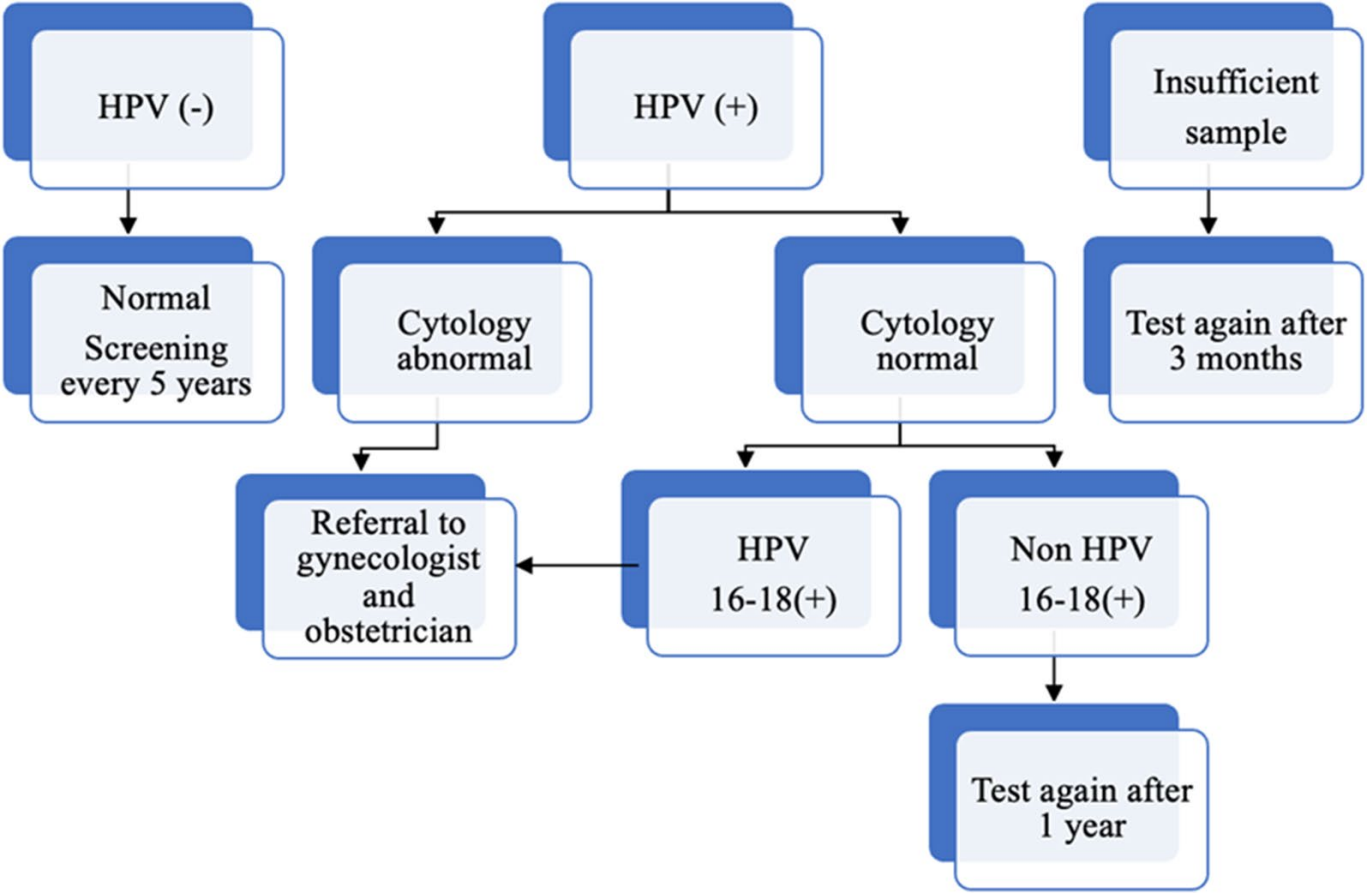
National Cervical Cancer screening program algorithm—Turkey

Screening tests available include HPV test, cytology (Pap smear test), and visual inspection with acetic acid [8]. It is thought that the HPV test will become the standard in cervical cancer screening in countries with different income sources in the coming years. Today, the cervical cancer screening program with the HPV test is performed in many countries [3]. In Turkey, a population-based cervical screening program using Pap smear was implemented in 2004, but it was unsuccessful due to the need for qualified professionals, lack of coordination infrastructure, quality assurance, and insufficient laboratory cytopathology capacity [9]. Today, in Turkey, according to national cancer screening standards, every woman in the 30-65-age group is screened every five years by HPV-DNA and Pap smear tests. In addition, these screenings are performed free of charge in KETEM (Kanser Erken Teshis, Tarama ve Egitim Merkezi; Cancer Early Diagnosis, Screening, and Education Centers), Family Health Centers (FHC), and Healthy Life Centers (HLC) [10]. The national cervical cancer screening program algorithm is presented in Fig. 1, and according to this, HPV-positive women with abnormal cytology or who are HPV 16- or 18-positive are referred to an obstetrician for further examination and treatment [11].

This study aimed to share the results of the national cervical cancer screening program conducted in primary health care institutions in Samsun.

## Methods

HPV DNA-based screening applied in our country started in 2014. Two national laboratories were established for the primary HPV screening program in Ankara and Istanbul. Samsun province samples are sent to the Ankara laboratory every Friday. The entire specimen testing is performed in the two laboratories by using fully automated operational procedures that allow tracing of specimens and timely delivery of results to the screened population. The Turkish Ministry of Health Cancer Control Department is responsible for the quality assurance and monitoring of the program. There is a standard working procedure with checkpoints for both HPV DNA analysis and the evaluation of pap smears, which are;

- Sampling adequacy: Inadequate sampling is monitored by four pathologists in the laboratory. Conventional cytology samples are double-blinded by at least two pathologists in at least 20% of the samples.
- Sampling Reports: Pap-smear samples of 10% of HPV-positive and normal cases are evaluated by the same four pathologists. The four pathologists working in the laboratories also evaluate each other’s reports. For this evaluation, about 10% of slides, which were reported as normal by one pathologist, are reviewed by the other to improve interobserver consistency and provide quality control targeting > 90% consistency in normal reports.
- HPV DNA analysis: Two control systems are used, internal and external. For internal control, one negative and one positive sampling are used for every 88 patients’ plates. For external quality control, re-evaluation is done in cooperation with UK National External Quality Assessment Service (UK NEQAS) at least twice a year, using ten external samples for validation [9].

The national software program developed for the HPV screening program RUNLEK ensures that it is monitored at every stage of the screening process. This system monitors all stages from distribution of HPV kits, transportation and collection, and recording of samples to reports. In case of any problem, it informs the authorized person in the laboratory via message and ensures that the problem is detected and solved.

d. Result reports: The screening results are reported online and sent to the family physician or the nurse at the center where the screening is performed. The medical staff can access the results themselves by logging in to the program. In addition, the person who has been screened can see her own report from this web-based system with her barcode number and citizenship number (https://hpvtarama.saglik.gov.tr/duyurular/sonucsorgula).

Results include the adequacy of the samples, HPV-positivity vs. negativity, HPV genotypes for positive cases, and cytological abnormalities for those with HPV-positivity [9].

As per the national cervical cancer screening standards, two samples are obtained from each woman included in the screening program; while the first sample is transferred onto the glass for traditional cytology, the second is evaluated in terms of HPV DNA analysis. The second sample is placed in 5 ml of Standard Transport Medium (STM) for HPV DNA analysis. For women who are HPV positive by Hybrid Capture2 (Qiagen), genotyping is performed with the CLART kit (Genomica). CLART kit (Genomica) is a polymerase chain reaction (PCR) based genotyping method and can detect 35 genotypes (16, 18, 31, 33, 35, 39, 45, 51, 52, 56, 58, 59, 68 and others). Thus, it is possible to study cytology in HPV-positive patients without the need to retake samples. Therefore, a more cost-effective approach can be possible, allowing resources to be used to increase cervical cancer attendance and follow-up of women with treatment and management needs. Samples of subjects with positive HPV DNA are evaluated in terms of cytology by two pathologists as double-blinded. The results reported as normal, infection, atypical squamous cells of undetermined significance (ASC-US), low-grade squamous intraepithelial lesions (LSIL), Atypical squamous cells-cannot exclude high-grade lesions (ASC-H), high-grade squamous intraepithelial lesions (HSIL), atypical glandular cells (AGC) in the cytological evaluation are again reported online to the center, in which scanning is performed, through a national software program created by the Ministry of Health. In this software program, whether the samples are sufficient, HPV positivity/negativity status, HPV genotypes for HPV-positive cases, and cytology reports are included [9, 12]. Women who with HPV 16/18-positivity or abnormal cytology are referred to an obstetrician for further examination and treatment (Fig. 1).

The research sample consisted of 316,675 women aged 30–65 living in Samsun in 2019. It was determined 89,302 (28.2%) women aged 30–65 were screened for cervical cancer between January 01, 2015, and December 31, 2019. The screenings were performed in 4 KETEM, 3 HLC, and 142 FHC serving in the city. The kits used in the program are provided by the Ministry of Health through a central tender and delivered to the screening centers through the health directorate. In our country, population information is recorded nationally with a standard software system. The appropriate target population list for screening comes to each family physician automatically through this software. Women in the target population are invited by their family physicians and other screening centers by phone or face-to-face interview. If there is no response to the invitation, the invitation is sent again every year. If there is no response after five years, women are registered in the system as a “rejection scan.“ Screening data are recorded with a web-based central system. The data were obtained by filtering the “National HPV Laboratory Application” automation program used by the Provincial Health Directorate Cancer Unit in April-May 2020 over the time of completion of the tests, and all results were evaluated without sampling (https://hpvtarama.saglik.gov.tr/hpvlab/Login?ReturnUrl=%2fhpvlab).

For the study, necessary permissions were obtained from Ondokuz Mayıs University Clinical Research Ethics Committee (OMU-KAEK 2020/79) and Samsun Provincial Health Directorate. Descriptive statistics of the data were expressed using mean ± standard deviation values and numbers (%). The Chi-square test was used in statistical analysis. Values of p < 0.05 were considered statistically significant.

## Results

In the study, the mean age of 89,302 women screened for cervical cancer between 2015 and 2019 was 45.9 ± 9.0 years. 46.9% of the women were in the 30–44 age group, 32.9% were in the 45–54 age group, and 20.2% were in the 55–65 age group. The women included in the screening program were analyzed regarding age groups. While the rate of women in the 30–44 age group was 43.0% in 2015, this rate was 47.5%, 46.8%, 46.6%, and 50.9% in the following years, respectively. 1.0% of the samples taken were considered insufficient samples, 94.1% as HPV negative, and 4.9% (4337 samples) as HPV-positive. The numbers of samples taken by year and positive test results are presented in Table 1.

**Table 1:** The number of samples by year and HPV positive test results

Year	Sample taken	Insufficient sample		HPV positive	
	n	n	%	n	%
2015	15,733	279	1.8	623	4.0
2016	24,328	206	0.8	1033	4.2
2017	18,313	163	0.9	912	5.0
2018	17,327	111	0.6	942	5.4
2019	13,601	101	0.7	827	6.1
Total	89,302	860	1.0	4337	4.9

Positive test results by years and age groups are presented in Table 2. The total HPV-positivity rate by the age groups was 5.7% (30–44 years old), 4.5% (45–54 years old), and 3.7% (55–65 years old), respectively. HPV-positivity rate was statistically significantly higher in the 30–44 age group (X^2^ = 122.725, *p* < 0.001) (Table 3). There were 7,865 different types of HPV genotypes in positive women. The most common HPV genotypes were 16, 51, 31, and 52 (Table 4).

**Table 2:** HPV positivity status by age groups

Year	30–44		45–54		55–65		Total	
	n	%	n	%	n	%	n	%
2015	312	0.8	217	0.7	94	0.5	623	0.7
2016	568	1.4	309	1.1	156	0.9	1033	1.2
2017	482	1.2	268	0.9	162	0.9	912	1.0
2018	512	1.2	268	0.9	162	0.9	942	1.1
2019	499	1.2	239	0.8	89	0.5	827	0.9

**Table 3:** Distribution of HPV test results by age groups

HPV test	30–44		45–54		55–65		Total		X^2^	p
	n	%	n	%	n	%	n	%		
Positive	2373	5.7	1301	4.5	663	3.7	4337	4.9	122.725	< 0.001
Negative	39,114	94.3	27,805	95.5	17,186	96.3	84,105	95.1		
Total sample*	41,487	100	29,106	100	17,849	100	88,442	100		

*Except for insufficient samples

**Table 4:** HPV genotypes among 4337 positive cases (7865 different types) by age groups

Genotype	30–44		45–54		55–65		Total	
	n	%	n	%	n	%	n	%
HPV16	660	15.5	330	13.9	139	11.4	1129	14.4
HPV18	169	4.0	94	4.0	42	3.4	305	3.9
HPV31	302	7.1	158	6.6	73	6.0	533	6.8
HPV33	87	2.0	64	2.7	37	3.0	188	2.4
HPV35	189	4.4	102	4.3	52	4.3	343	4.4
HPV39	170	4.0	89	3.7	40	3.3	299	3.8
HPV45	104	2.4	66	2.8	24	2.0	194	2.5
HPV51	324	7.6	151	6.3	100	8.2	575	7.3
HPV52	245	5.7	145	6.1	83	6.8	473	6.0
HPV56	149	3.5	96	4.0	50	4.1	295	3.7
HPV58	151	3.5	93	3.9	34	2.8	278	3.5
HPV59	144	3.4	88	3.7	43	3.5	275	3.5
HPV68	160	3.8	90	3.8	59	4.7	309	3.9
Other	1409	33.1	813	34.2	447	36.5	2669	33.9
Total	4263	100	2379	100	1223	100	7865	100

Considering the cytology results of HPV-positive 4337 women, 441 (10.2%) were considered as inadequate, 3236 (74.7%) (including infection 36.5%) as negative, 308 (7.1%) as ASC-US, 298 ( 6.9%) as LSIL, 20 (0.5%) as ASC-H, 10 (0.2%) as HSIL, and 24 (0.6%) as AGC (Table 5). Of 660 (15.1%) women with abnormal cytology findings, 46.7% were ASC-US, 45.2% were LSIL, 3.6% were AGC, 3.0% were ASC-H, 1.5% were HSIL. It was determined that the most common subtype in HPV-positive women was HPV 16/18 (n = 1379, 31.7%). When the smear results of women with HPV 16/18 were examined, 143 (10.3%) specified as having insufficient samples were excluded from the evaluation, and the remaining 992 (80.3%) were considered as negative (including infection, 41.1%) and 244 (19.7%) as abnormal. The number of cytology samples considered abnormal was 416 (14.0%) in women with oncogenic types other than HPV 16/18 (n = 2958).

**Table 5:** Distribution of cytology results of HPV-positive women

Cytology results	n	%
Inadequate materials	441	10.2
Negative	3236	74.7
ASC-US	308	7.1
LSIL	298	6.9
ASC-H	20	0.5
HSIL	10	0.2
AGC	24	0.6
Total	4337	100

As per these results, 1795 (2.0%) women who were HPV-positive and had abnormal cytology or were HPV 16/18-positive, even if the cytology was normal, were referred to a specialist physician (Fig. 1). As per the Training and Research Hospital data, a tertiary hospital designated as a reference diagnosis center by the Samsun Health Directorate, data of 1603 (89.3%) women referred from screening centers between 2015 and 2019 were reached. It was determined that 1352 of these women underwent colposcopy, and a biopsy was taken from 1072. When the biopsy results were examined, 417 (38.9%) women were found to have normal test results, while 425 (39.6%) women had pre-invasive (CIN 2-CIN 3) lesions, 209 (19.5%) had other results (LSIL; ASCUS), and 17 (1.6%) had cancer. 4 (0.4%) of the samples were considered insufficient.

## Discussion

There is a risk for women that HPV infection may become chronic and pre-cancerous lesions progress to cervical cancer. Sexually active women should be screened for abnormal cervical cells and pre-cancerous lesions, starting from 30 years of age [13]. The HPV screening program, initiated in our country in 2014, also starts in this age group and is performed by family physicians. When country data are analyzed, the number of women participating in the screening program has been observed to increase in the following years [9]. According to the data of our province, although the number of women screened varies over the years, the positivity may have increased over the years due to the younger age group being included in the screening. In addition, the fact that family physicians repeatedly invite women on their screening lists as required by the legislation and national campaigns may cause an increase in the positivity rates over the years. Considering the 5-year screening results in our study, the HPV-positivity rate was found as 4.9%, and the abnormal cytology rate was 15.2% in the positive ones.

Today, HPV-based screening test replaces cytology because it is more effective in protecting against cervical cancer, provides long screening intervals, and is less costly [14, 15]. WHO has also made a global call to highlight the importance of screening with vaccination as part of a triple intervention strategy to eradicate cervical cancer [16].

In many countries such as the United States, Australia, Netherlands, Italy, Norway, and Sweden, HPV testing is applied for screening purposes [17–19]. HPV-positivity may indicate different results as per the regions. In a study where the first results of the cervical cancer screening program conducted in our country were shared, the HPV-positivity rate was found to be 3.5%, and the rate of abnormal cytology was 19.1% [9]. Considering the HPV-positivity in different countries, it is 8.1% in the screening performed in Australia and 9.9% in the general population in the Asian continent [20, 21]. While it ranges from 43.8 to 55.8% in different studies conducted in Kazakhstan, it was 22.49% in a study conducted in China and 19.7% in the Caribbean region [22–24]. These differences in the geographical distribution of HPV-positivity can be associated with many factors such as socio-cultural differences in societies, perception of risky behavior, and sexual experience at an early age. It is stated that 70–80% of sexually active women are infected with HPV, usually shortly after sexual activity begins [25]. Therefore, sexual behaviors such as active sexual life, unprotected sexual intercourse, and polygamy increase the risk of being infected with HPV. HPV positivity in our study decreases with age, similar to other studies, and when evaluated in terms of oncogenic HPV types, these subtypes are more commonly encountered at younger ages [9, 20, 24]. We think this may be related to early and frequent sexual intercourse.

In our study, the rate of HPV 16/18, considered the oncogenic type in HPV-positive women, was 31.7%. This rate was 33.22% in the results of Gültekin et al.‘s study [9]. Moreover, in the study, it was stated that in developing countries such as Turkey, HPV 16–18 genotyping is sufficient to refer the patients to colposcopy in terms of both cost and human resources. While the risk of developing squamous cell carcinoma of the cervix is low in women who are not infected with HPV, this risk increases 250–400 times in those infected [26]. It is also thought that this genotyping will meet the need for epidemiological mapping and contribute to shaping policies related to HPV prevention, vaccination, and screening with detailed data.

When the Pap smear results of HPV-positive women in our study were examined, 15.1% had abnormal cytology. The rate of abnormal cytology in HPV-positive women was 8.8%, 24.4%, and 30.4% in studies conducted in different cities in our country [27–29]. In our study, when HPV-positive women were evaluated in terms of abnormal cytology results, the ASC-US and LSIL results were very close, respectively. Afterward, the results were listed as AGC, ASC-H, and HSIL. Looking at the country results, LSIL was first and then ASC-US and HSIL, respectively [9]. Since various risk factors (such as multiparity, oral contraceptive use, smoking, and immunosuppression) are known to increase the risk of HSIL in women infected with HPV, we think that the difference is due to this situation [30]. It is remarkable that in 484 (35.0%) of 1379 women who participated in the screening program and were HPV 16/18 positive, the cytology result was normal, suggesting that HPV scanning is more effective than cytology, similar to the literature [17, 31, 32].

WHO global call stressed that every country should not reach the goals of 90-70-90 by 2030 in order to embark on the path of eliminating cervical cancer in the next century [6]. The HPV vaccine is currently used in many countries, and it is thought that the results of the national screening program will lead the studies on the application of this vaccine in our country. Various studies are performed to increase the coverage rates in countries implementing screening programs, and studies are also designed on self-HPV sampling. In these studies, it is stated that self-sampling is beneficial in removing obstacles to screening performed by the clinician [33–36]. In studies of our country, the gynecological examination is seen as one of the obstacles to screening for cervical cancer. [37–39]. The study of Sözmen et al. revealed that the number of women participating in cancer screening in our country is low [38]. Studies in different regions have shown that the barriers to cervical cancer screening programs are similar. Among them, embarrassment, physical privacy anxiety, fear of pain, fear of cancer, and worrying about what the test will find are among the first places. Lack of information is also a major obstacle. In a study conducted on Muslim women, in addition to similar obstacles, social support, short processing time, and responsibility to protect one’s own health due to religious belief were found as facilitating reasons [40–44].

Because it is retrospective, we could not determine why women in the screening group did not accept screening in our study. Considering that we are a country with a majority Muslim population, it is thought that religious belief and cultural structure, together with obstacles in other studies, have an impact on this. Situations such as lack of information, not being aware of health services, or having difficulty in accessing them may be among other reasons due to the geographical structure of our city. For this reason, awareness activities should be planned according to regional characteristics and aim to reach all segments of the society especially through primary health care personnel. In addition, we think that the self-test method will be useful for achieving the desired target in the number of scans.

Our study has some limitations. One of them is that there were no demographic variables (such as educational status, occupation, chronic disease) other than women’s age since the data were obtained from the automation program. Another limitation was that not all follow-up results of women who participated in the screening program and were HPV-positive could be reached. Besides, another was that it was only performed on women admitted to health institutions, and the number of women included in screening programs was low.

## Conclusion

Our study determined that women who were included in the screening program in Samsun and who had abnormal HPV and Pap smear results were detected. However, it is necessary to reduce insufficient sample rates and include more women in the screening program. Identifying people who are HPV-positive and providing early diagnosis and treatment will decrease the number of cancer cases as well as the deaths from this cancer. These studies will also gain favor in improving women’s health. Hence, community participation should be ensured by carrying out awareness studies on screening programs, and the effectiveness and inclusiveness of cancer screening programs should be increased across the country.

## Data Availability

The datasets generated and/or analyzed during the current study are not publicly available due to this type of use not being included in the written consent form but are available from the corresponding author on reasonable request.

## Abbreviations

HPV: Human Papilloma Virus
WHO: World Health Organization
CIN: Cervical intraepithelial neoplasia
KETEM: Kanser Erken Teshis, Tarama ve Egitim Merkezi (Cancer Early Diagnosis, Screening, and Education Centers)
FHC: Family health centers
HLC: Healthy life centers
ASC-US: Atypical squamous cells of undetermined significance
LSIL: Low-grade squamous intraepithelial lesions
ASC-H: Atypical squamous cells-cannot exclude high-grade lesions
HSIL: High-grade squamous intraepithelial lesions
AGC: Atypical glandular cells
